# FoxM1 Promotes Cell Proliferation, Invasion, and Stem Cell Properties in Nasopharyngeal Carcinoma

**DOI:** 10.3389/fonc.2018.00483

**Published:** 2018-10-26

**Authors:** Weiren Luo, Fei Gao, Siyi Li, Lei Liu

**Affiliations:** ^1^Department of Pathology, Department of Scientific Research and Education, The Second Affiliated Hospital of Southern University of Science and Technology, Shenzhen Third People's Hospital, Shenzhen, China; ^2^Guangdong Provincial Key Laboratory of Cancer Immunotherapy Research, Cancer Research Institute, Southern Medical University, Guangzhou, China; ^3^Department of Gastroenterology, The First Affiliated Hospital of Jinan University, Guangzhou, China; ^4^Gastroenterology Research Unit, Division of Gastroenterology and Hepatology, Department of Physiology and Biomedical Engineering, Enteric Neuroscience Program, Mayo Clinic, Rochester, MN, United States

**Keywords:** FoxM1, forkhead box protein M1, stem cell, proliferation, nasopharyngeal carcinoma, pathology—head and neck neoplasms

## Abstract

**Background:** The self-renewal and tumourigenicity of FoxM1 in nasopharyngeal carcinoma (NPC) remain largely unknown. In this study, we attempt to investigate the self-renewal and tumourigenicity of FoxM1 and its clinical significance in nasopharyngeal carcinoma (NPC).

**Methods:** Several assays including cell counting Kit-8 (CCK-8) assays, colony formation, flow cytometry, immunofluorescence, tumor spheres, and mice model were used to detect the biological function of FoxM1 in NPC. The association between FoxM1 and clinical pathological features, and stem cell markers was analyzed using immunohistochemistry.

**Results:** High expression of FoxM1 was prominently present in the T4 stages, cancer cells migrating into the stroma and vasculature. Overexpression of FoxM1 enhanced tumor proliferation, cell cycle progression, migration and stress fibers formation *in vitro*. In NPC tissues, FoxM1 correlated significantly with stem cells-related clinical pathological features including late clinical stage, tumor recurrence and distant metastasis. Meanwhile, FoxM1 linked closely with the expression levels of stem cell markers including Nanog, Sox2, and OCT4 in tumor samples, and also promoted the expression of these stemness-related genes *in vitro*. Moreover, FoxM1 conferred the self-renewal properties of cancer cells by increasing side populations (SP) cells and formed larger and more tumor spheres. Importantly, FoxM1 enhanced the ability of tumourigenicity of NPC cell lines in mice xenograft.

**Conclusions:** We demonstrate that FoxM1 greatly induces cancer progression and cancer stem cell (CSC) features in NPC.

## Introduction

Nasopharyngeal carcinoma (NPC) is a head and neck cancer that is endemic in Southern China and Southeast Asia (1). A majority of patients diagnosed with advanced stages III or IV, will have a poor outcome (2, 3). Nevertheless, most patients die because of the development of local relapse and metastasis after therapy. However, the precise mechanisms accounting for its high-aggressive behaviors remain largely unclear.

Cancer stem cells (CSCs) are defined by a subpopulation of cells within tumors have the capability of self-renewal and generating new tumors, are responsible for cancer unlimited proliferation, invasion, and late stage of patients (4, 5). Forkhead box (FOX) superfamily of proteins play an important role in regulating a wide range of transcriptional activities ranging from cell homeostasis and development (6). FoxM1 is pro-oncogene transcription factor contributing greatly to cell cycle progression where endogenous FoxM1 expression regulating expression of a variety of G2/M-specific genes (7, 8). Conversely, suppression of FoxM1 results in reducing cancer cell proliferation, migration and metastasis. Interestingly, FoxM1 has an integral role in cancer initiation and cancer drug response (9, 10), indicating that FoxM1 might be a new and effective therapeutic target for the elimination of CSCs. For example, Hou et al. reported that the depletion of FOXM1 sensitized the resistant cells to paclitaxel treatmente by regulating the gene transcription of abcc5 in NPC cell lines (10). However, up to now, the roles of FoxM1 in the tumorigenesis and stem cell-like features in NPC remain largely unknown.

This study is to investigate the roles of FoxM1, by examining its regulation and the effect of FoxM1 overexpression or knockdown in NPC cells, and further detected in cancer biopsies. Our data represent the strong indication that FoxM1 directly contributes to caner progression and stem cell-like properties in NPC.

## Materials and methods

### Cell culture

NPC cell lines (6-10B and SUNE1 with low metastatic abilities and 5-8F with high metastatic potential) were cultured in RPMI1640 medium (Life Technologies, Carlsbad, CA) containing 10% fetal bovine serum (FBS) at 5% CO_2_ and 37°C in a humidified incubator.

### Clinical samples

Tumor tissues including 113 paraffin-embedded NPC tissues and 29 nasopharyngitis tissues were obtained from in the Department of Pathology, Guangdong Medical College, China (2003–2005). All of patients had no received radiotherapy/adjuvant chemotherapy. Informed consent was obtained from the Institutional Research Ethics Committee. The clinicopathologic features of NPC samples were shown as previously described (11, 12).

### Plasmids, lentivirus production and transduction

Lentiviral expression clones for FoxM1 (LV-pGV208-FoxM1) or a control vector (LV-pGV208), and the lentiviral packaging plasmids (pHelper 1.0 and pHelper 2.0) were bought from Genechem (Shanghai, China). According to the manufacturers' instruction. Virus supernatant was harvested from these cells after 48 h transfection, and subsequently used to infect 6-10B and SUNE1 cells. To attain 100% percentage of infected cells, lentiviral infected cells were sorted by Flow Cytometry based on EGFP assay. The successful overexpression of FoxM1 was verified by qRT-PCR and Western blot. The FoxM1 siRNAs were purchased from Genechem (Shanghai, China). siRNAs (working concentration of 100 nmol/L) were transiently transfected into cells using Lipofectamine 2000 reagent (Invitrogen) in accordance with the operating manual.

### qRT-PCR

In brief, Trizol Reagent (TaKaRa) was used to extract total RNA from NPC cells according to the production protocol. In this method, total RNA was reversely transcribed by using the PrimeScript RT reagent Kit (TaKaRa). Expression of mRNA analysis was done using SYBR Green Master Mix (TaKaRa). For the amplification of genes, the primers in this study were used as previously described (8, 13). The experimental cases were normalized to internal controls and fold changes were analyzed by 2^−ΔΔ*Ct*^ quantification.

### Western blot

The cell proteins were resolved on 10% SDS-polyacrylamide gels, and electrophoretically transferred to polyvinylidene difluoride (PVDF) membranes (Millipore). After transfer, the membranes were incubated with antibodies followed by HRP-labeled goat-anti-mouse or rabbit IgG and detected by chemiluminescence. The blots were incubated with the primary antibodies against abbit-anti-FoxM1, Nanog, Oct4 and Sox2 (Abcam). Mouse-anti-ABCG2 (Santa Cruz Biotechnology), Mouse-anti-MMP1, MMP9 (BD Biosciences). The hybridization signal was observed using enhanced chemiluminescence (ECL). GAPDH was considered as an internal control.

### Immunofluorescence analysis

For phalloidin assay to detect F-actin cytoskeleton, the cells were placed on culture slides firstly (Costar, MA). After 24 h, the cells were washed with PBS and fixed in 4% paraformaldehyde for 10 min, and then permeabilized with triton X-100 (0.05%). Next, the cells were blocked for 30 min with 10% BSA (Sigma, MO) and then incubated with 200 nM working stock of Acti-stain™ 670 phalloidin for staining the actin cytoskeleton in cells. Cell nuclei were counterstained with 4-,6-diamidino-2-phenylindole (DAPI; Sigma, St. Louis, MO) for 5 min, and imaged with a confocal laser-scanning microscope (Olympus FV1000).

### Immunohistochemistry

The procedure of IHC was performed as previously described (11, 12). The slides were incubated overnight at 4°C with primary antibodies as bellow: Rabbit-anti-FoxM1, Nanog, Oct4, and Sox2 antibodies were purchased from Abcam (Cambridge, UK). Mouse-anti-ABCG2 (Santa Cruz Biotechnology, CA.). IHC staining was examined and scored by two independent pathologists without knowing the clinical characteristics. PBS was used as blank controls.

### Cell proliferation and colony formation assays

A Cell Counting Kit-8 (CCK-8) was used to determine cell proliferation rates according to the manufacturerprotocol (Dojindo Laboratories, Kumamoto, Japan). Experiments were performed in triplicate. In brief, 1 × 10^3^ cells/well was seededin 96-well culture plates. The cells were incubated with the solution for l h, then optical density (OD) was calculated at 450 nm. For cell formation assay, cells were seeded in 6-well culture plates (500 cells/well). The culture medium was renewed every 3 days. After 2 weeks, the colonies were fixed with methanol and stained with 0.1% crystal violet. Colonies more than 50 cells were counted.

### Cell cycle analysis

The cells were placed onto the 6-well plates (1 × 10^6^ cells/well) and fixed with 70% cold ethanol at 4°C overnight. The cells were incubated in 1 ml of cellular DNA staining solution (20 mg/mL propidium iodide; 10 U/mL RNaseA) at room temperature for 30 min after being washed with PBS for three times. The DNA content of labeled cells was collected by FACS caliber flow cytometry (BD Biosciences). The assay was done in triplicate.

### Tumor spheres formation assay

Briefly, single cells were digested with 0.25% trypsin (Sigma, St. Louis, MO) and suspended in serum-free medium (DMEM-F12 50 ml+ 100 μg/ml EGF+100 μg/ml bFGF+B27 supplement 1 ml). The cells (1,000 cells/ml) were seeded on ultra-low attachment plates (Corning, Corning, NY, United States). After 5~14 days, cells spheres were counted under microscope.

### Sorting of SP cells by flow cytometry

As previously described (14), tumor cells were digested using 0.25% trypsin (Sigma, St. Louis, MO), washed for two times with calcium/magnesium-free PBS, and then resuspended in ice-cold RPMI 1640 culture (supplemented with 2% FBS) at a dose of 1 × 10^6^ cells/mL. Further, Hoechst 33342 (Sigma, St. Louis, MO) was added (5 mg/mL) and the cases were incubated in dark with periodic mixing for 70–90 min at room temperature. After beingwashed twice with PBS, 1 mg/mL propidium iodide (Sigma, St. Louis, MO) was added, and the samples were put at 4°C in dark before sorting by flow cytometry (BD FACSAria).

### Nude mice xenograft assay

Female BALB/c nude mice (4–5 weeks) were bought from the Medical Laboratory Animal Center of Guangdong Province. All experiments were approved by the Ethics of Animal Experiments of the Southern Medical University. Three mice per group of nude mice were underwent subcutaneous injection of 100 μl of FoxM1-overexpressing and control cells at doses of 10^4^ and 10^6^, respectively. Tumors of each group were photographed after 6 weeks of tumor growth. Individual tumors were fixed and embedded in 10% paraffin to assess tumor pathology. The expression of markers (FoxM1, Ki67, and BrdU) were analyzed by IHC in each tissue.

### Statistical analysis

All data were analyzed using SPSS standard version 13.0 (SPSS, Chicago, USA). The χ2-test was used to assess the relationship between the clinical features and FoxM1 expression. The data were presented as mean ± SEM from at least 3 independent experiments. Two-tailed Student's *t*-test was performed for the comparisons with 2 independent groups. ^*^P < 0.05 and ^**^P < 0.01 were considered as statistical significance.

## Results

### FoxM1 promotes tumor carcinogenesis in nasopharyngeal carcinoma

**Figure 1:**
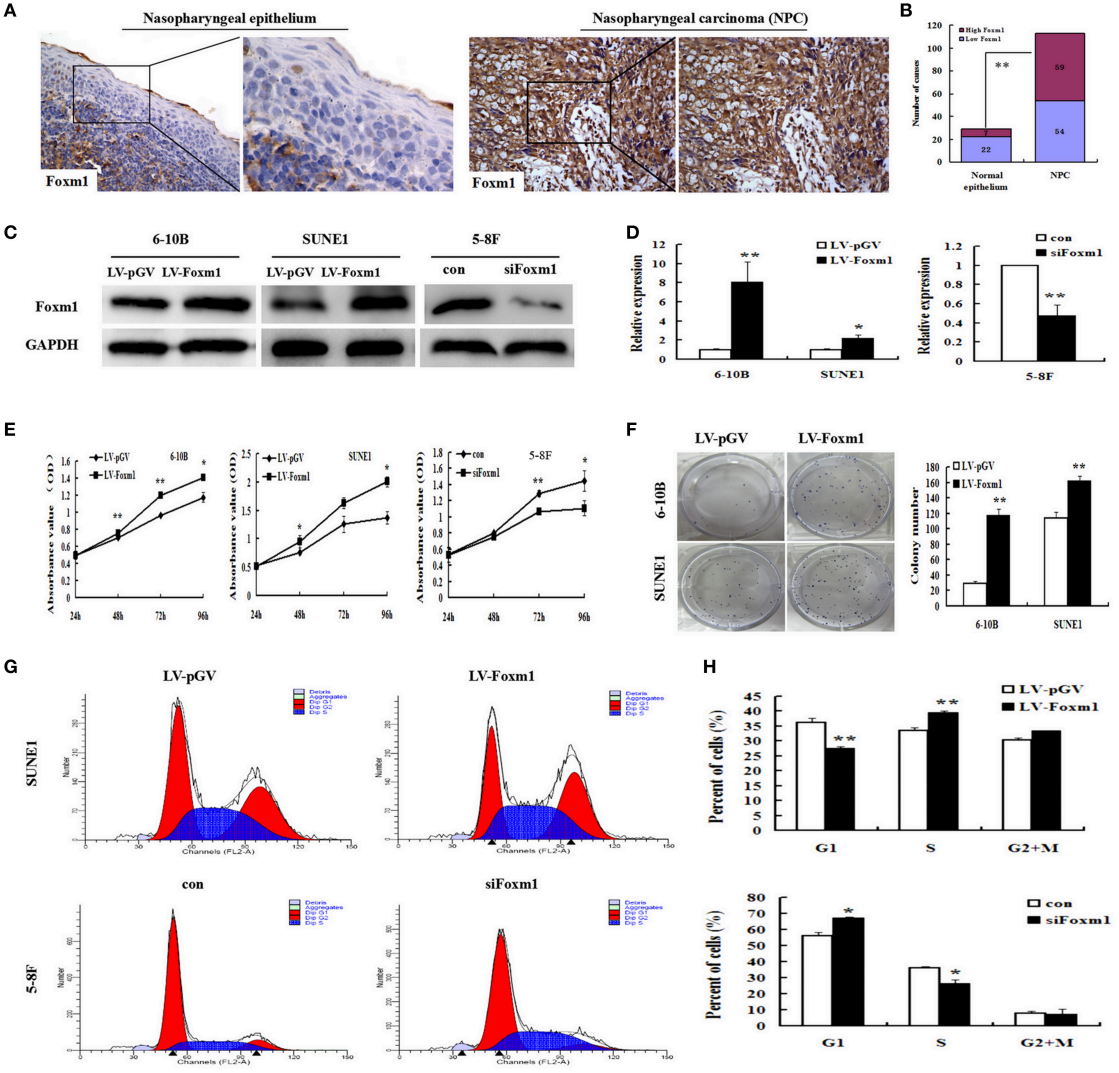
FoxM1 promotes tumor carcinogenesis and proliferation in nasopharyngeal carcinoma (NPC) **(A,B)**. FoxM1 expression in NPC and non-cancerous nasopharyngeal tissues based on IHC. Weak FoxM1 expression in non-tumoral pharynx epithelium and strong staining in NPC samples **(C,D)**. The expression of FoxM1 in 6-10B and SUNE1 cells carrying FoxM1 transgene and 5-8F with stably FoxM1 knockdown by qRT-PCR and Western blot, respectively **(E)**. The cell growth rates were determined by CCK8 assay **(F)**. Colony formation of 6-10B and SUNE1 cells with or without exogenous FoxM1 expression. The assay was performed in triplicate **(G,H)**. The cell cycle distribution in overexpressing-FoxM1 SUNE1 and siFoxM1 5-8F cells by FACS. Histograms showing the changes of G1/S phase S phase in SUNE1 and 5-8F cells after overexpressing and knockdown FoxM1 gene, respectively. ^*^P < 0.05; ^**^P < 0.01.

IHC staining was used to detect the expression levels of FoxM1 protein in 113 NPC tissues and 29 non-cancerous nasopharyngeal samples. FoxM1 was found to be localized in the nucleus and cytoplasm of primary cancer cells. FoxM1 protein was weakly detected in the non-cancerous nasopharyngeal epithelium. However, among the 113 samples, High expression of FoxM1 was detected in 59 cases (Figures 1A,B). FoxM1 was significantly upregulated in the tumor tissues compared with the non-tumor tissues, indicating FoxM1 was involved in the carcinogenesis of NPC (*P* < 0.01).

To clarify the effects of FoxM1 upregulation in the pathogenesis of NPC, FoxM1 stably overexpressed 6-10B and SUNE1 cells were generated. Levels of FoxM1 transgene in three kinds of NPC cells were tested by qRT-PCR and Western blot. As listed in Figure 1D, the levels of FoxM1 gene in 6-10B and SUNE1 cells harboring FoxM1 transgene were significantly higher than that in cells vector control (~ 8 and 2.5-folds) using qRT-PCR (*P* < 0.01). The results were verified by Western blot (Figure 1C).

We found that cell proliferation was significantly increased in FoxM1-overexpressing 6-10B and SUNE1 cells in comparison with vector-control cells by the CCK-8 assay (Figure 1E). In contrast, cell proliferation was significantly inhibited in siFxom1 5-8F cells than the vector-control group. These data indicates that upregulation of FoxM1 increased cell proliferation ability in NPC cells. According to colony assay (Figure 1F), exogenous expression of FoxM1 also significantly increased the colony formation of 6-10B and SUNE1 cells (*P* < 0.01). In contrast, the colony formation of knockdown FoxM1 was significantly decreased in 5-8F cells than control groups (47 ± 6 vs. 132 ± 7; *P* < 0.01). Fluorescence-activated cells sorting (FACS) was used to analyze the role of FoxM1 on cell proliferation of NPC. We found that FoxM1-overexpressing SUNE1 cells had 27.89% of G1 phase cells, which was lower than 37.23% of G1 phase cells in control cells. However, 40.69% of the SUNE1-FoxM1 cells were in the S phase, which was higher than the control cells (33.26%) (Figures 1G,H; *P* < 0.01). These findings imply that FoxM1 accelerated SUNE1 cells from G0/G1 phase into S phase. In contrast, FoxM1 depletion attenuated 5–8F cells from G0/G1 phase into S phase.

### FoxM1 contributes to tumor motility in NPC

**Figure 2:**
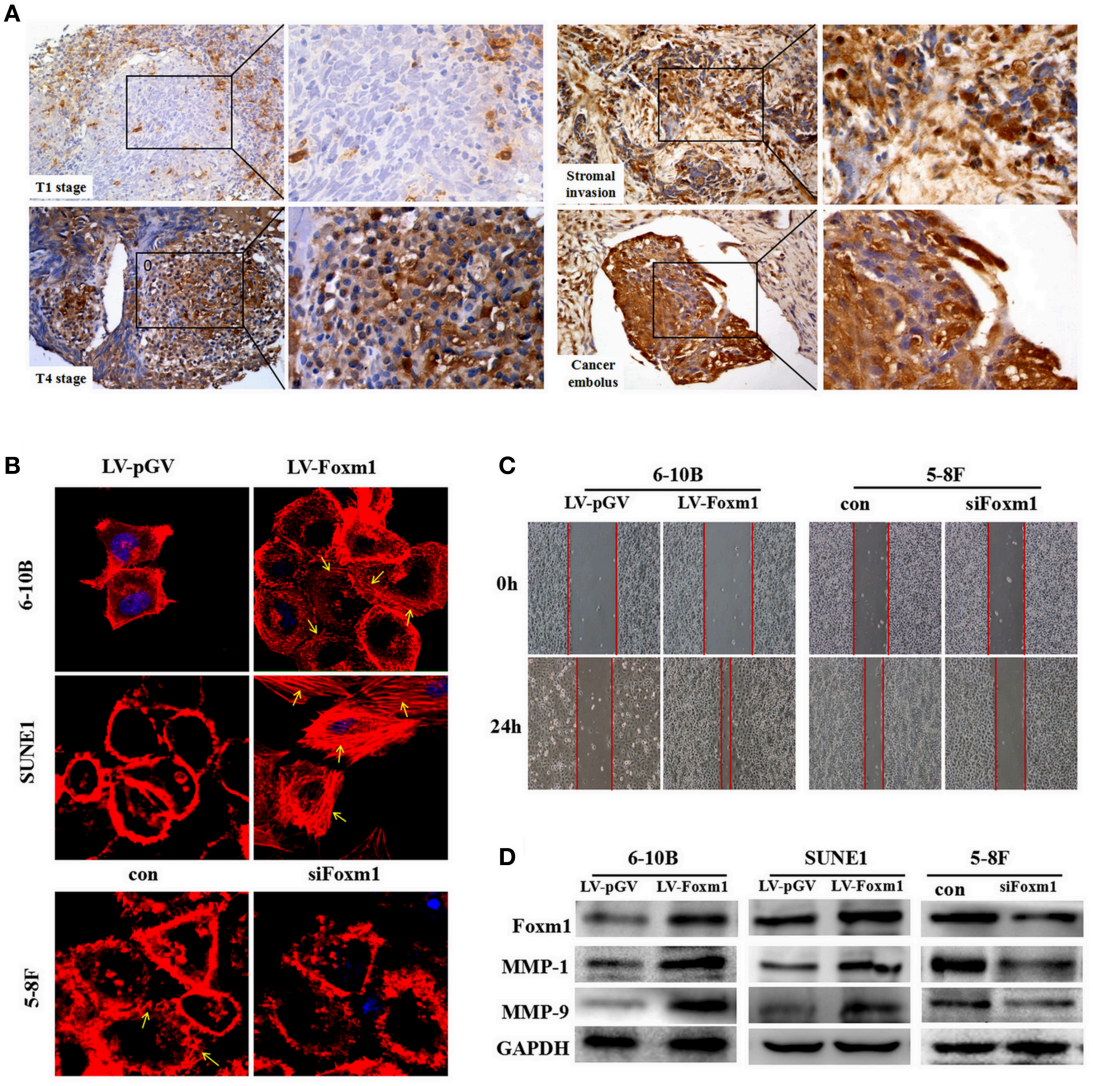
FoxM1 confers tumor migrative behaviors in NPC **(A)**. Representative images of high expression of FoxM1 in the T4 stages, cancer cells migrating into the stroma and vasculature **(B)**. Cytoskeleton (stress fibers formation) is measured by fluorescein-labeled phalloidin (red). Stress fibers formation is especially present in the leading edges (arrows indicated) **(C)**. FoxM1 accelerates the migration of both 6-10B and SUNE1 cells surveyed by scratch migration assay **(D)**. Western blot illustrates that FoxM1 increases expression of MMPs including MMP1 and MMP9, whereas FoxM1 knockdown has the opposite effect.

Firstly, we found that overexpression of FoxM1 correlates with aggressive behaviors in NPC tissues. In 113 cases, FoxM1 expression was positively associated with tumor infiltration. High expression of FoxM1 was detected in patients with T4 stages than that in T1 stage (Figure 2A, *P* < 0.01). Additionally, FoxM1 expression was found highly increased in the cancer cells migrating into the surroundings and vasculature (Figure 2A).

Next, the effect of FoxM1 on tumor migration was tested. As shown in Figure 2C, the scratch migration assay revealed that FoxM1-expressing 6-10B and SUNE1 cells displayed significantly increased mobility than control cells (*P* < 0.01). In addition, the stress fiber formation (stained with phalloidin stain) was predominantly occurred in FoxM1-expressing cells, especially in the leading edges, but not in LV-pGV-expressing cells (Figure 2B). Furthermore, overexpression of FoxM1 caused the increased expression levels of MMP1 and MMP9 (Figure 2D).

### FoxM1 correlates with stem cells-related clinical pathological features in NPC

**Figure 3:**
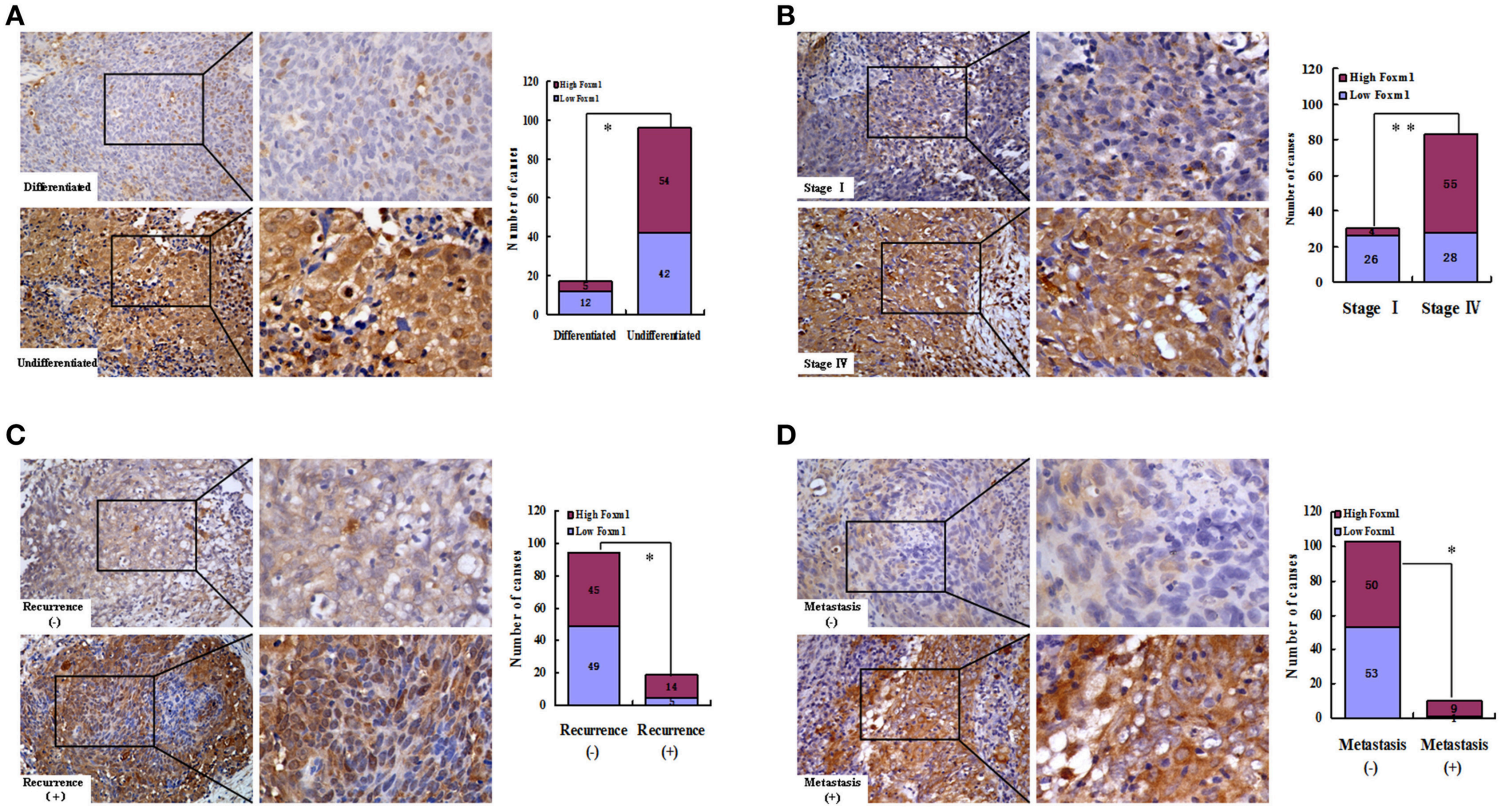
FoxM1 links with cancer stem cells (CSCs)-related clinical pathological features in NPC. Representative images of high expression of FoxM1 was observed in the undifferentiated **(A)**, stage IV**(B)**, tumor recurrence **(C)** and distant metastasis **(D)** stages of NPC biopsies. ^*^P < 0.05; ^**^P < 0.01.

We found that FoxM1 protein was weakly expressed in differentiated type of NPC (differentiated nonkeratinizing carcinoma, DNKC). However, among the 96 samples of undifferentiated type (undifferentiatied carcinoma, UDC), high expression of FoxM1 was found in 54 cases (Figure 3A; Table 1). As listed in Figure 3B, overexpression of FoxM1 was only found in 4 of 30 patients with stage I, whereas 55 cases were observed with high FoxM1 expression among 83 samples of stage IV (*P* < 0.01). Moreover, FoxM1 expression was found significantly more common in tumors with local recurrence (*P* = 0.040) and distant metastasis (*P* = 0.012) compared with those with no recurrence or metastasis (Figures 3C,D). There was a statistically significant relationship between FoxM1 expression and histologic subtype (*P* = 0.042), clinical stage (*P* = 0.000), tumor recurrence (*P* = 0.040) and distant metastasis (*P* = 0.012) (Table 1). These findings indicate that high expression of FoxM1 might confer stem cell-like characteristic during NPC progression.

**Table 1:** Correlation of FoxM1 overexpression with clinical characteristics in 113 NPCs.

**Variables**	***N***	**FoxM1 expression**			**FoxM1 expression**	
		**Low (*N*, %)**	**High (*N*, %)**	***P***	**Spearman correlation**	***P***
**SEX**						
Man	86	45 (52.3)	41 (47.7)	0.085	0.162	0.086
Woman	27	9 (33.3)	18 (66.7)		
**AGE (y)**						
< 50	55	29 (52.7)	26 (47.3)	0.306	0.096	0.310
≥50	58	25 (43.1)	33 (56.9)		
**TUMOR HISTOLOGY**						
DNKC	17	12 (70.6)	5 (29.4)	0.041	0.192	0.042
UDC	96	42 (43.8)	54 (56.2)		
**T CLASSIFICATION**						
T1-T2	54	35 (64.8)	19 (35.2)	0.001	0.326	0.000
T3-T4	59	19 (32.2)	40 (67.8)		
**N CLASSIFICATION**						
N0-N1	63	39 (61.9)	24 (38.1)	0.001	0.317	0.001
N2-N3	50	15 (30.0)	35 (70.0)		
**M CLASSIFICATION**						
No	103	53 (51.5)	50 (48.5)	0.012	0.236	0.012
Yes	10	1 (10.0)	9 (90.0)		
**TUMOR RECURRENCE**						
No	94	49 (52.1)	45 (47.9)	0.040	0.193	0.040
Yes	19	5 (26.3)	14 (73.7)		
**TUMOR STAGE**						
I-II	30	26 (86.7)	4 (13.3)	0.000	0.468	0.000
III–IV	83	28 (33.7)	55 (66.3)		

DNKC, differentiated nonkeratinizing carcinoma; UDC, undifferentiated carcinoma; T, tumor size; N, lymph node metastasis; M, distant metastasis

### FoxM1 increases stemness properties of NPC *in vitro*

**Figure 4:**
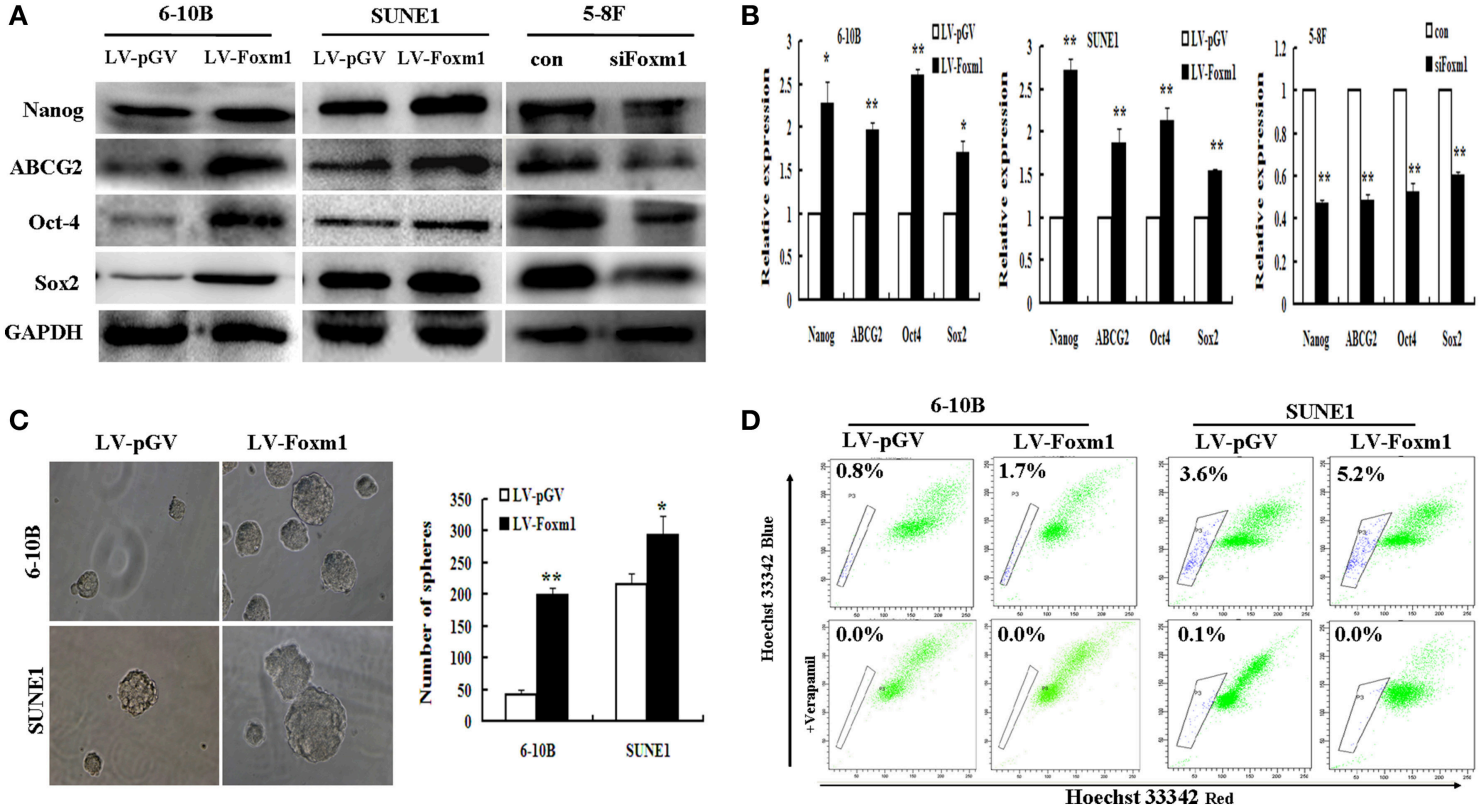
FoxM1 induces stem-related gene expression and properties in NPC **(A)**. Western blot reveals an increased expression of stem cells markers Nanog, ABCG2, OCT4, Sox2 in FoxM1-expressing cells, and decreased expression in the knockdown group **(B)**. qRT-PCR demonstrates an increased expression of Nanog, ABCG2, OCT4, Sox2 in FoxM1-expressing cells, while inhibited in the knockdown control **(C)**. FoxM1 induces stem cell-like self-renewal properties. Sphere sizes (left panels), the numbers of spheres (right panels) **(D)**. FoxM1 increases the size of the side population (SP) cells. Flow cytometric profiles of SP cells among the 6-10B and SUNE1 cell lines after stable expression of FoxM1. ^*^P < 0.05; ^**^P < 0.01.

Consequently, the association between stable expression of FoxM1 and stem cell-like phenotype was explored in NPC cell lines. Stem cell markers mentioned above were performed by RT-PCR and Western blot. As shown in Figure 4A, FoxM1 up-regulateed the protein levels of the stem cell markers including Nanog, ABCG2, Oct4, and SOX2 compared with the control (LV-pGV), whereas FoxM1 knockdown significantly attenuated the expression levels of these proteins. The increasing of Nanog, ABCG2, Oct4, and SOX2 were further confirmed at the transcriptional level (Figure 4B).

Additionally, the effect of FoxM1 on the capacity of cancer stem cells to self-renew was examined. Tumor sphere formation assay displayed that FoxM1 overexpressing cells form larger and more spheres compared with control cells (Figure 4C). We further determined whether the expression of stem cell markers and increased tumor spheres were caused by an increase of SP cells. As shown in Figure 4D, the stable expression of FoxM1 dramatically increased the size of the SP in the 6-10B (from 0.8 to 1.7%) and SUNE1 (from 3.6 to 5.2%) cell lines in comparison with the control group (*P* < 0.01).

### FoxM1 promotes tumor initiating capacity of NPC *in vivo*

**Figure 5:**
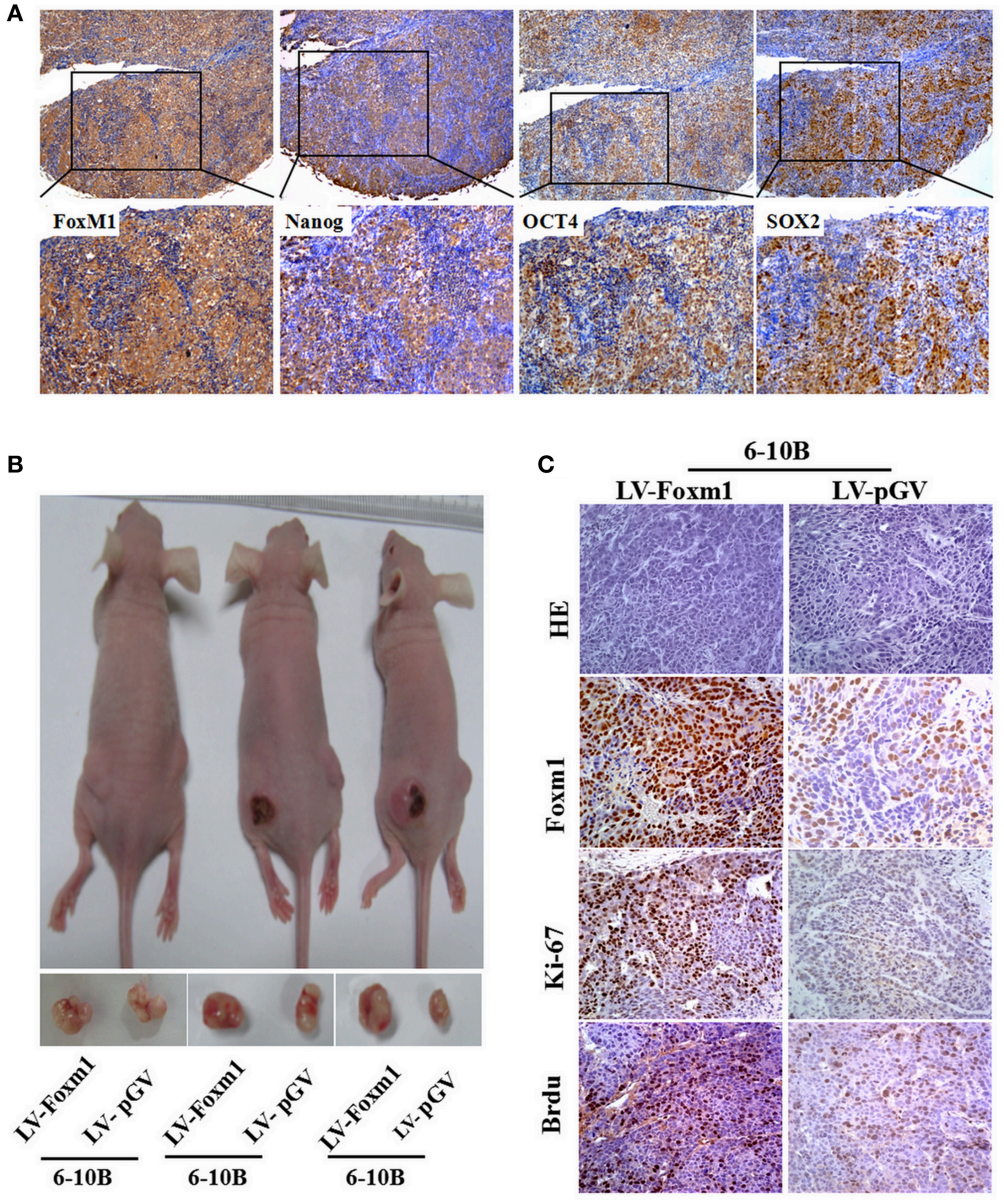
FoxM1 enhances the tumorigenesis of NPC *in vivo* **(A)**. A significant association between the expression of FoxM1 and stem cell-like markers including Nanog, OCT4, Sox2 in a series of NPC sections **(B)**. Representative images of xenograft tumors in nude mice inoculated with 6-10B cells stably expressing FoxM1 or vector control (three groups) **(C)**. H&E staining and IHC analysis of FoxM1, Ki67, and BrdU expression of cell lines stably expressing FoxM1 and control in nude mice.

To determine the correlation between FoxM1 and the expression of stem cells markers Nanog, SOX2 and OCT4 in NPC samples, the expression of these proteins was examined in 113 NPC biopsies by IHC. We found a positive association between FoxM1 and Nanog (*r* = 0.650; *P* = 0.000), SOX2 (*r* = 0.218; *P* = 0.020), and OCT4 (*r* = 0.243; *P* = 0.010) expression in NPC tissues (Figure 5A). These data strongly imply that patients with high FoxM1 expression might have more tumor initiating characteristics. As mentioned above, SP cells and stem cell-like features were found to be enriched in FoxM1-overexpressing cells, the effects of FoxM1 on the tumourigenicity of NPC were evaluated in animal models. Tumors formed after FoxM1 or vector control expressing 6-10B cells were injected in nude mice. We found that the injection of FoxM1-overexpressing cancer cells into nude mice yielded larger tumors than in the control groups (Figure 5B). The terminal tumor weights were increased significantly compared with the control groups (0.52 ± 0.06 vs. 0.28 ± 0.08, *P* < 0.05). On the other sides, the final tumor volumes were increased greatly compared with the control groups (436 ± 69 mm^3^ vs. 223 ± 58 mm^3^, *P* < 0.05). This indicates that FoxM1-overexpressing cells had a higher tumorigenic ability than control cells. Furthermore, the expression levels of FoxM1, Ki67, and BrdU was investigated through IHC in primary xenograft tumors. We found that higher expression of FoxM1, Ki67, and bromodeoxyuridine (BrdU) in the FoxM1-overexpressing tumors compared with the controls (Figure 5C). Moreover, stem cell markers Nanog, ABCG2, Oct4, and SOX2 were assessed by IHC and qRT-PCR. Compared with the control (LV-pGV), FoxM1 up-regulates the stem cell markers Nanog, ABCG2, Oct4, and SOX2 at the protein level. Taken together, FoxM1 might increase the ability of tumor-initiating cells in NPC.

## Discussion

The overexpression of FoxM1 has been observed in a broad range of human cancer biopsies including breast cancer, colorectal cancer and prostate cancer, suggesting that FoxM1 is essential for caner proliferation and carcinogenesis (5, 6). Compared with the non-cancer epithelium, we also found that FoxM1 protein was upregulated in NPC tissues, indicating FoxM1 might be involved in the carcinogenesis of NPC. According to the CCK-8 assay and colony assay, exogenous expression of FoxM1 significantly increased the growth and colony formation of 6-10B and SUNE1 cells, indicating that FoxM1 can promote cell proliferation ability in NPC cells. To investigate the effects of FoxM1 protein on cell proliferation of NPC, FACS was further performed to investigate the cell cycle. We found that up-regulation of FoxM1 facilitated the transition of SUNE1 cells from G0/G1 into S phase. On the other side, several reports demonstrate interfering FoxM1 transcription factor can suppress cell proliferation and induce apoptosis of various cancers including NPC by down-regulation of cyclin D1 and E1 (15–17). For example, Jiang et al. reported that FoxM1 inhibition by thiostrepton or siRNA transfection induced cell apoptosis of NPC cells by down-regulation of bcl-2, up-regulation of bax and p53. These data strongly confirm the notion that FoxM1 protein is as a key regulator of cancer cell proliferation.

Cell invasion have been an important characteristics of cancer progression and CSCs (18). Interestingly, we found that FoxM1 was highly exhibited in patients with T4 stage in comparison with those in T1 stage. This observation indicate that tumor cells with FoxM1-overespressing in NPC might have high motility to migrate into the surroundings. Actually, overexpression of FoxM1 was also observed in cancer cells migrating into the surroundings and vasculature. These results suggest that FoxM1 might be crucial for aggressive behaviors of NPC patients. Indeed, scratch migration assay showed that FoxM1-expressing 6-10B and SUNE1 cells possessed strongly increased mobility. As reported, cytoskeletal reorganization is a prerequisite for cell motility (19). In this study, we found an increase and reunion of cytoskeleton in FoxM1-overexpressing NPC cells. Accumulating evidence has demonstrated that FoxM1 can increase the invasive properties of tumor cells via matrix metalloproteinases (MMP) signaling pathway (20, 21). As we know, MMPs is crucial for the invasion and metastasis of tumor cells (22, 23). However, the close relationship between FoxM1 and MMPs expression still remains unclear in NPC. Here we shows that protein expressions of MMP2 and MMP9 were greatly enhanced by FoxM1 expressing in 6-10B and SUNE1 cells, suggesting a novel role of FoxM1 in regulating migration of NPC cells.

Cancer stem cells (CSCs) are responsible for cancer tumorigenesis, local recurrence, and metastasis (9, 10). Firstly, we found that FoxM1 protein was more frequently present in the undifferentiated type of NPC than in the differentiated type. Supporting our results, it has provided evidence that undifferentiated counterparts of several solid tumors (including lung adenocarcinoma and breast cancer) confer higher proportions of stem cell-like properties (24–26). Additionally, for the first time, we demonstrated that FoxM1 expression correlated closely with local recurrence and distant metastasis in NPC patients. As we know, local recurrence and distant metastasis contribute to poor prognosis of NPC patients. Supporting our findings, Huang et al. reported that FOXM1 overexpression predicted a poorer overall survival of NPC (27). Nanog, Sox2, and OCT4, the critical biomarkers of embryonic stem cells (ESCs), have been shown to correlate strongly with the properties of CSCs (28–30). Our previous results reveal that the high expression of SOX2, OCT4, and Nanog played an important role on tumor aggressiveness and poor prognosis of NPC (31). In this study, FoxM1 linked strongly with the expression of these stem cell markers (Nanog, Sox2, and OCT4) in NPC samples, and also enhanced the expression of stem-related genes *in vitro*. In a whole, we propose that FoxM1 might determine and maintain a distinct subpopulation possessing stem cell-like features in NPC.

As far as we know, FoxM1 could lead to CSC phenotype in several cancers including ovarian cancer, breast cancer, colon cancer, pancreatic cancer and glioblastoma (7, 32–34). However, the effect of FoxM1 on “stemness” in NPC remains to be characterized. Side population (SP) cells are derived from stem cells of many types of cancers, and these cells share characteristics of CSCs and enrich tumor initiating capacity (35). Wang et al. has demonstrated that that SP cells in NPC cell lines had stem-like characteristics and were enriched for tumor initiating ability in nude mice (14). We also reported that aldehyde dehydrogenase 1(ALDH1), a novel marker for isolating CSCs in human NPC, are more frequently present in the SP cells (36). These results show that SP cells might be enriched for stem cell characteristics in NPC. To further explore whether the expression of FoxM1 could induce stem cell features in NPC, the effects of FoxM1 on the capacity of self-renewal as SP cells were examined. As result, we observed that the FoxM1 expression strongly promoted the proportion of the SP in the 6-10B and SUNE1 cell lines in comparison with the control group. On the other hand, tumor sphere, which is derived from human cancer cells and samples, can self-renew and produce tumors in mice (37). Here, we also showed that FoxM1 overexpressing cells formed larger and more tumor spheres. To our knowledge, high tumorigenic capability in animal models is the gold standard for the definition of CSCs. Our study found that the FoxM1 tumor cells grew at a higher rate than the control groups in all examples. Furthermore, BrdU label-retaining cells were observed to be highly present in FoxM1-overexpressing cells of xenograft tumors. In 2007, Yao et al. has demonstrated firstly that the presence of epithelial BrdU label-retaining cells was considered as stem cells in human NPC samples, which are most likely responsible for NPC metastasis and local recurrence (38). Based on these results, we propose the idea that FoxM1 induces stem-like cell self-renewal and tumourigenicity in NPC. It has shown that FOXM1 regulates stemness and survival of cancer cells via several pathways. For example, Hamurcu et al. found that FOXM1 regulates expression of eukaryotic elongation factor 2 kinase and promotes proliferation and tumorgenesis of human triple negative breast cancer cells (39). The molecular mechanisms that underlie the role of FoxM1 in NPC stemness need to be further elucidated.

## Conclusion

In summary, all combined results from this study and other groups suggest a novel role for FoxM1 in cancer progression and stem cell features in NPC. Further insights into the exact role of FoxM1 are needed by elucidating the underlying signaling network that regulates the FoxM1 pathways.

## Ethics statement

This approach was approved by Human Research Ethics Committee of Guangdong Medical University. All subjects gave written informed consent according to the Declaration of Helsinki.

## Author contributions

WL and FG participated in the design of the study. WL performed the critical revision of the manuscript for important intellectual content. WL and FG performed the data collection and analysis, interpreted the data. WL and SL produced the main draft of the manuscript and made figures. WL and LL obtained funding for the study.

### Conflict of interest statement

The authors declare that the research was conducted in the absence of any commercial or financial relationships that could be construed as a potential conflict of interest.
